# First-line therapy in HER2 positive metastatic breast cancer: is the mosaic fully completed or are we missing additional pieces?

**DOI:** 10.1186/s13046-016-0380-5

**Published:** 2016-06-30

**Authors:** Alessandra Fabi, Paola Malaguti, Sabrina Vari, Francesco Cognetti

**Affiliations:** Division of Medical Oncology 1, Regina Elena National Cancer Institute, Via Elio Chianesi 53, 00144 Rome, Italy

**Keywords:** HER2 target terapies, Pertuzumab, Trastuzumab, Metastatic breast cancer

## Abstract

The discovery of human epidermal growth factor receptor 2 (HER2) and its role in the biology of breast cancer and the subsequent development of HER2-targeted therapies, have dramatically improved clinical outcomes for women with early-stage and advanced HER2-positive breast cancer (BC).

HER-2 targeted therapies represent a major step forward in achieving the goal of delivering individualized targeted therapy for BC, and trastuzumab was the first anti-HER-2 strategy to be approved for treatment of HER-2 positive BC.

This review discusses the treatment of metastatic HER2-positive BC and describes efficacy and safety of novel anti-HER2 target therapies in first-line metastatic settings and the future challenges include refining such treatments, reducing toxicity and simultaneously developing innovative therapies. Furthermore, combinations of trastuzumab and drugs targeting the downstream pathway are described.

In the next future will be possible to use an ample armamentarium of combination therapies directed against HER2 and key signaling components integrated in the HER network. This approach will allow clinicians to tailor the management of the individual patient on the basis of tumor- specific biomarker profiles.

There is an urgent need for prospective biomarker-driven trials to identify patients for whom targeting is cost-effective.

## Background

About 20 % of invasive breast cancers (BC) are HER2-positive and characterized by amplification and/or overexpression of HER2, a transmembrane receptor with tyrosine kinase (TK) activity, resulting in HER2 gene amplification on chromosome 17. This subtype of BC shows an intrinsic malignancy and has a poor prognosis in the absence of specific treatment. About 10 % of the cases will be diagnosed as metastatic disease and the 5-year overall survival (OS) in these patients achieves 20 % (historical median survival range 16–29 months), of which 2–5 % are long-term survivors [1, 2] On the other hand HER2 espression identifies patients who may benefit from anti-HER2 targeted therapies [3–11].

Trastuzumab, a humanized monoclonal antibody that selectively binds to the HER2 on the surface of tumour cells, when added to chemotherapy in the first-line advanced treatment of HER2-positive BC is associated with a significantly reduced risk of progression and death compared to chemotherapy alone [12–16].

However, despite these deep therapeutic advances, the progression free survival (PFS), and OS of patients treated with trastuzumab and chemotherapy-based regimens for metastatic BC (MBC) is about 7 months (vs about 5 months without trastuzumab) and 25 months (in contrast to 20 months without trastuzumab) respectively [17, 18].

In the last 5 years we are witnessing an exponential development of new anti HER2 molecules and results of their activity in terms of outcome for the advance tumor are very exciting.

In this article, we will analyze the most relevant aspects related to new developments with anti-HER2 therapies and the more recent clinical evidences and therapeutic strategies with the modern use of biological anti HER2 drugs at the first progression of BC patients.

## HER2: the beginning of the story

With regard to blocking gene expression, the most significant strategy in HER2-targeted therapies have been made using monoclonal antibodies directed against the extracellular domain of the HER2 protein, named trastuzumab. Binding with high affinity to the extracellular domain of HER2, trastuzumab inhibits the proliferation of tumour cells that overexpress HER2. It demonstrated antitumour effects when administered as a single agent, and additive and synergistic effects when administered in combination with a range of antineoplastic agents (1).

After the first phase II trials in which it was underlined clinical efficacy of trastuzumab-including chemotherapy (anthracyclines, paclitaxel) at the first recurrense of disease, the successive evolution in clinic was to compare chemotherapy alone versus a combination of chemotherapy plus trastuzumab as first-line therapy in MBC patients whose tumors overexpressing HER2 [17–19]. The addition of trastuzumab to chemotherapy significantly increased overall response rate [ORr (50 *vs.* 32 %, *p* < 0.001)], duration of response [DR (9.1 *vs.* 6.1 months, *p* < 0.001)], time to progression [TTP (7.4 *vs.* 4.6 months, *p* < 0.001)], time to failure [TTF (6.9 vs 4.5 months, *p* < 0.001)]. The addition of trastuzumab to paclitaxel increased the ORr from 17 to 41 % (*p* < 0.001), the DR from 4.5 to 10.5 months (*p* < 0.01), the TTP from 3.0 to 6.9 months (*p* < 0.001) and the TTF from 2.9 to 5.8 months (*p* < 0.001). The addition of trastuzumab to adriamycin plus cyclophosphamide (AC) increased the RR from 42 to 56 % (*p* = 0.02), the DR from 6.7 to 9.1 months (*p* = 0.005), the TTP from 6.1 to 7.8 months (*p* < 0.001) and the TTF from 5.6 to 7.2 months (*p* < 0.001). The median OS for patients who received trastuzumab and chemotherapy combination was 25 months *vs.* 20 months for those who received chemotherapy alone (*p* = 0.046). This result is even more significant in light of the fact that two-thirds of the women who are progressive on chemotherapy alone arm went on to receive open-label trastuzumab on a compassionate-use protocol [18]. Therefore, the magnitude of the survival advantage may have been diminished due to this crossover bias. The quality of life (QoL) was also studied using standardized QoL questionnaires.

Overall, there was a statistically significant improvement in fatigue and global QoL in the chemotherapy plus trastuzumab group [20]. As seen in previous experiences, patients with immunoistochimical (IHC) 3+ staining appeared to benefit more from trastuzumab than those in the IHC 2+ subgroup. Furthermore, significant benefit was seen only in patients whose tumors were positive for gene amplification by fluorence in situ hibridation (FISH) [21]; in these subgroups of patients the OS was improved with the use of trastuzumab combined to chemotherapy (Odds Ratio 0.71, 95 % CI:0.54 to 0.92; *p* = 0.009) compared to chemotherapy alone. No such differences were noted in the FISH negative subgroup. It must be kept in mind, however, that these results were derived from a retrospective subset analysis, and the number of FISH-negative subjects was small (*n* = 108). Based upon the results of these phase II and III trials, trastuzumab was approved for use in MBC patients with HER2-amplified or overexpressed as monotherapy (second line or later) or in combination with paclitaxel (first-line) by the United States FDA in September 1998.

Other combinations showed to be effective such as docetaxel plus trastuzumab [16, 22] and vinorelbine plus trastuzumab [23].

## Multi-targeted HER2 agents: the new frontiers

Additional therapies targeted to other HER2 pathways to be used in combination with trastuzumab are being explored both in adjuvant and metastatic settings [24, 25].

HER2-targeted treatment can be used as single- (e.g. trastuzumab, pertuzumab and trastuzumab-MCC-DM1) or multi-targeted HER2 agents (e.g. pertuzumab, lapatinib) combined with standard sistemic treatment (chemotherapy or hormone therapy, e.g. taxane, anthracyclines, anastrozole, letrozole).

With regard to the management of advanced HER2-positive BC, trastuzumab, pertuzumab, trastuzumab emtansine (formerly known as T-DM1) and lapatinib are approved as standard care for inhibiting HER2 activity in the treatment of HER2 positive MBC and for increasing the incidence of PFS, OS and overall response rate (ORr) when compared with chemotherapy alone or standard anti-HER2 molecule [26–28, 29–31]. But to date, only the combining of trastuzumab and pertuzumab have indication in the first-line setting.

## The first recurrence in HER2 positive MBC patients: what do we offer today?

What to do and what not to do at the first recurrence in HER2 positive MBC patients?

One important study showed us what not to do for first-line MBC: the MA-31 trial compared lapatinib/taxane followed by lapatinib vs trastuzumab/taxane followed by trastuzumab in 600 patients; it found a PFS detriment for lapatinib/taxane at the interim analysis [31]. Moreover, this combination showed an increased febrile neutropenia and grade 3 or higher grade of diarrhea. We need to remember these data in clinical decision-making when lining up our therapeutic options for women at the firse appearence of HER2 positive disease.

The survival bar has now been raised to over 15 months with the combination of pertuzumab plus trastuzumab. The CLEOPATRA study [26, 32] showed the big power of adding a second antibody, pertuzumab, to trastuzumab as first-line metastatic therapy. Pertuzumab, binds to HER2 receptor but at a different site to trastuzumab, and is able to inhibit ligand-induced dimerization of HER2 with its receptor partners [33]. Preclinical experiments showed that pertuzumab and trastuzumab produced a more-complete blockade of the HER signaling network when combined, and were more effective in HER2-positive tumor xenografts, than either antibody alone [34].

The study assessed the efficacy and safety of three-weekly pertuzumab (fixed loading dose of 840 mg iv., followed by 420 mg iv.) with trastuzumab (8 mg/kg iv., followed by a maintenance dose of 6 mg/kg iv.) plus docetaxel (administered at a starting dose of 75 mg/m^2^; at the discretion of the investigator, the dose could be increased to 100 mg/m^2^ if the side-effect profile was acceptable), compared with placebo plus trastuzumab and docetaxel (at the same doses). The final findings resulted in significant reduction in the risk of progression or death and an increase of 6.1 months in median PFS (18.5 vs 12.4 months, *p* < 0.001).

The CLEOPATRA trial changes the clinical practice. We now have a new standard of care for advanced disease espressing HER2. In any country of the world, for patients with HER2 positive MBC the proposal for treatment should include dual targeting with pertuzumab and trastuzumab plus docetaxel or paclitaxel. The findings of this study are really outstanding, because showed a median OS of 56.5 months that is unprecedented in first-line MBC with an improvement in terms of OS of 15 months respect to trastuzumab alone.

## Who were the patients of Cleopatra study?

If we analyze the demographic characteristics of the patients treated in Cleopatra study [32], we notice that there is no difference in terms of results among patients who had previously received adjuvant/neoadiuvant chemotherapy and the patients with de novo metastatic disease. Although the number of patients who received prior neo-adjuvant treatment with trastuzumab was small, the benefit in terms of PFS achieved with double block plus docetaxel in this population was similar to that observed in subgroup not receiving previous neo- or adjuvant therapy (*p* = 0.36).

Another intriguing aspect is the age of Cleopatra’s patients: the median age was 54 years (range 27–89); in the subgroup analysis by older age (>65 or <65) all the patients showed a benefit in terms of PFS and OS in favour of the dual block. In patients over 75 years no significant difference were observed (*p* = 0.76).

With regard to the biological characteristics of the disease, both in triple positive tumors (expression of both hormonal receptors [HRs] and HER2) and HER2-like tumors (not expression of HRs and HER2 positivity), all patients obtained a clinical benefit in terms of PFS and OS in dual targeting arm, but the greatest benefit is expressed in the HER2-like disease [0.61 (0.47–0.81)].

Also the visceral disease seems to get a better PFS, but the number of patients with non-visceral disease was very limited, and therfore no defined considerations it is possible to drive.

The safety profile of pertuzumab, trastuzumab and taxane was consistent with the known safety of patients with long-term exposure to dual targeting. It means that we now have a treatment that improves both PFS and OS without affecting the QoL of patients in terms of haematological, not haematological and cardiac safety. This information is very important for the long duration of exposure to the two biological molecules which the patient can support.

## Blockade of HER2 and not only

Constitutive activation of PI3K/AKT/mTOR signalling due to PTEN loss can lead to trastuzumab resistance. mTOR inhibition sensitises PTEN-deficient tumours to trastuzumab, thereby suggesting that the combination of everolimus, the mTOR inhibitor, and trastuzumab have a role in the treatment of HER2-overexpressing BC [35, 36].

The addition of the mTOR inhibitor, everolimus, to trastuzumab and chemotherapy showed clinical benefit in heavily pretreated patients with HER2-positive MBC progressing on previous trastuzumab and taxane therapy [37].

No so high enthusiasm was declared when the two target combination was tested in first-line MBC. In Bolero 1 trial, patients were randomly assigned to receive either everolimus 10 mg once a day orally or placebo plus weekly trastuzumab intravenously (4 mg/kg loading dose on day 1 with subsequent weekly doses of 2 mg/kg of each 4 week cycle) and paclitaxel intravenously (80 mg/m2 on days 1, 8, 15 of each 4 week cycle) [38].

This study showed that the addition of everolimus to trastuzumab plus paclitaxel did not improve clinical outcomes. In the entire population, median PFS was 15 months (95 % CI: 14.55-17.91) with everolimus arm versus 14 months with placebo arm (HR 0.89, 95 % CI:0.73-1.08; *p* = 0. 11). In the HRs negative subpopulation, median PFS with everolimus arm was 20 months (95 % CI: 14.9-24.0) versus 13 months (10.0-16.5) with placebo arm (HR 0.66, 95 % CI: 0.48-0.91; *p* = 0.004); There was a clinically relevant prolongation of PFS with the addition of everolimus in HRs negative patients (7 months), but the p value did not meet prespecified criteria for significance by a small margin. However, based on the statistical design, the threshold for statistical significance in the HRs-negative subpopulation was rather stringent (*p* = 0.0044) [39]. This result in the HRs-negative patients are consistent with the previously reported predefined subgroup analysis in BOLERO-3 wherein patients with HRs negative and HER2 positive MBC derived more benefit when everolimus was added to HER2- targeted treatment in the absence of hormonal therapy [38].

Two ongoing phase 1 trials are assessing the benefits of adding PI3K/mTOR inhibitors to endocrine therapy and HER2-targeted therapy as first-line treatment in patients with both HRs and HER2-positive advanced BC. Mayer and colleagues are investigating the combination of an α-specific PI3K inhibitor BYL719 plus letrozole and trastuzumab (NCT01791478) and Wheler and colleagues are assessing everolimus plus letrozole and trastuzumab (NCT02152943). Further investigations of these compounds in the HR-negative sub-population are also of interest.

## What about T-DM1 in the first-line treatment?

Despite the substantial activity of trastuzumab, resistance to therapy arises in the majority of patients with advanced disease, so there is a continued need to develop novel agents to target HER2-amplified progressive disease.

Evidence that HER2-directed therapies are most effective when combined with cytotoxic chemotherapy, led researchers to develop a novel antibody–drug conjugate, T-DM1, in which highly potent maytanisinoid chemotherapy is stably linked to the HER2-targeted monoclonal antibody, trastuzumab [40, 41].

Recently, T-DM1 has been approved by the European Medicines Agency (EMA) and by the Food and Drug Administration (FDA), as a single agent for the treatment of patients with HER2-positive locally advanced and unresectable or MBC, who have previously received taxane- and trastuzumab-based treatment, separately or in combination [42].

The mechanism of action of this molecule is very exciting. Once T-DM1 binds to the extracellular domain of HER2, the complex is internalized into the cell, where the antibody is degraded by proteases, releasing the active metabolite, lysine-Ne -MCC-DM1, into the cytoplasm. Because this metabolite is a charged molecule, it is relatively membrane impermeable, reducing the possibility that the DM1 could enter a neighboring cell, thus further limiting the potential for nonspecific toxicity. In addition to its ability to deliver DM1 selectively to tumor cells, T-DM1 retains the effector functions of trastuzumab, including inhibition of HER2-mediated signal transduction and activation of antibody-dependent cellmediated cytotoxicity. Therefore, T-DM1 through this mechanism, leads to a double antitumor effect, an anti-HER2 effect mediated by the trastuzumab activity and a selective transport of a powerful antimitotic agent DM1 to the intracytoplasmic area. This innovative and selective mechanism of action increases the efficacy while, at the same time, it reduces the toxicity [42].

In preclinical experimental studies, T-DM1 induces a cell-cycle arrest and apoptosis in HER2 positive cancer cells but has little effect on HER2-negative cells. T-DM1 demonstrates activity in both trastuzumab and lapatinib-resistant HER2 positive cancer models [43, 44].

The evidence of clinical activity of T-DM1 in HER2 MBC was demonstrated in the phase III EMILIA trial [45] in which pretreated patients with one or two lines of trastuzumab-including chemotherapy were assigned to receive either lapatinib (1250 mg daily continuously p.o.) plus capecitabine (1000 mg/m2 p.o. twice a day, 14 days on, 7 days off) or T-DM1 every 3 weeks. The primary end point was achieved with the median PFS of 9.6 months in the T-DM1 group versus 6.4 months in the lapatinib plus capecitabine group (HR 0.65; 95 % CI: 0.55–0.77; *P* = 0.001). Median OS crossed the stopping boundary for efficacy and was increase by 5 months in the T-DM1 group (31 months versus 25 months; HR 0.68; 95 % CI, 0.55–0.85; *P* = 0.001). Secondary endpoints including ORr (43.6 % versus 30.8 %; *p* = 0.001) and median DR (12.6 months versus 6.5 months) favored T-DM1. In addition, the median time to decrease of 5 points or more in the Functional Assessment of Cancer Therapy-Breast (FACT-B TOI) score was delayed in the T-DM1-treated patients (7 months versus 4.6 months; HR 0.80; 95 % CI:0.67–0.95; *P* = 0.012). A further analysis of patients who had not a first-line treatment demonstrated a significant additional benefit in terms of PFS in favor of the new molecule (10.3 vs 6.7 months).

In February 2013, after EMILIA results, T-DM1 was approved by FDA as a treatment for HER2 positive pre-treated MBC.

The next step after Emilia results was to evaluate T-DM1 in the first line choice. A phase 2 randomized trial (TDM4450g) compared three-weekly T-DM1 to three-weekly docetaxel plus trastuzumab in 137 patients with previously untreated HER2 positive MBC [46]. Patients in the control group received trastuzumab plus docetaxel (75 or 100 mg/m^2^, per investigator choice). The ORr (58 % in the control group versus 64 % in the T-DM1 group; *p* = 0.458) and clinical benefit rate were similar in the two treatment groups; however, T-DM1 was associated with a significant 5 months prolongation in median PFS (9 months versus 14 months; HR: 0.59; 95 % CI: 0.36–0.97). Interestingly, there was a much lower rate of grade 3/4 serious adverse events with T-DM1 group occurring in 58 % of patients in the control arm group compared with only 6 % in the T-DM1 group. In the docetaxel/trastuzumab group, the most common toxicities were alopecia, neutropenia, diarrhea, and fatigue and more patients used colony-stimulating factors; a very different adverse events occorred in TDM-1 group, such as fatigue, nausea, elevated serum aspartate aminotransferase, fever and headache. A lower rate of adverse events in the T-DM1 group translated to an improvement in quality of life [46].

Unfortunately, patients experience disease progression on T-DM1; hence, discovering molecular markers that can predict response or resistance to this molecule remains a high priority.

The results from combination of T-DM1 + pertuzumab in synergistic inhibition of tumor cell line proliferation in vitro, provided the rationale for further study.

In MARIANNE trial [39], 1095 patients with progressed or recurrent locally advanced or previously untreated metastatic HER2-positive BC were assigned to receive T-DM1 plus pertuzumab, T-DM1 plus placebo or trastuzumab plus docetaxel/paclitaxel (control arm). At the time the trial was initiated, the control arm represented the standard of care for this patient population. After a median follow-up of 35 months, both T-DM1–containing regimens showed noninferior PFS, but not superiority, over control arm. The median PFS was 15 months in the T-DM1 plus pertuzumab arm (HR: 0.87, 95 % CI: 0.69-1.08; *p* = 0.14), 14 months with T-DM1 alone (HR 0.91, 95 % CI :0.73-1.13; *p* = 0.31) compared with 13.7 months with trastuzumab and taxane. The OS data were not yet reached. The objective response rate was 64 %, 60 %, and 68 % among the three schedules, respectively. However, the median duration of response was 21 months both in the T-DM1 plus pertuzumab arm and in the T-DM1 alone arm, and 12.5 months in the control arm. This unexpected result has induced researchers to review projects of trials in course.

So, for the time being, Cleopatra regimen remains the preferred first-line therapy for HER2-positive MBC, and the treatment guidelines for 2016 remain unchanged.

## Vertical blockade in first-line: is there a position for it?

Dual inhibition targeting both intracellular and extracellular domain of HER2 has also been tested in metastatic HER2 positive BC, as demonstrated by preclinical studies in HER2-positive cell lines, that shown a synergistic interaction between lapatinib and trastuzumab [47].

In the phase III EGF104900 trial, a heavily pretreated patients which had progressed on prior trastuzumab-based regimens was randomly assigned to receive combination of lapatinib and trastuzumab or lapatinib monotherapy [48]. In the intent-to-treat population who received a median of three prior trastuzumab-containing regimens, the combination of lapatinib with trastuzumab versus lapatinib alone significantly improved PFS (HR: 0.73; 95 % CI: 0.57-0.93; *P* =0.008) and clinical benefit rate (25 % in the combination arm versus 12 % in the monotherapy arm; *p* = 0.01), thus offering a chemotherapy-free option with an acceptable safety profile to patients with HER2-positive MBC. In the final survival analysis, dual HER2 blockade led to significant 4.5-months improvement in OS (HR: 0.74, 95 % CI: 0.57–0.97, *P* = 0.026) in the HER2 positive MBC [48]. A small cohort of untreated HER2 positive patients for metastatic disease received Chemotherapy-free treatments as in first-line treatment. Median OS was 43 months and median persistence on protocol was 3.8 months, with 4 patients (21 %) persisting on single agent for longer than 12 months. The gene expression analysis revealed that high expression of the 17q12-21 amplicon genes HER2 and GRB7, and the PAM50 HER2-enriched intrinsic profile, were significantly associated with longer persistence on protocol. Conversely, high expression of luminal-related genes such as PGR, MDM2 or PIK3CA, or the PAM50 luminal intrinsic profile correlated with reduced persistence on protocol. Moreover, increasing H2T/p95 ratio was found to be significantly associated with longer persistence on protocol (HR 0.56 per 2-fold increase in H2T/p95, *p* = 0.0015) [49].

The data suggest that patients belonging to the “HER2-enriched” subtype and/or having high H2T/p95 protein expression ratio are exquisitely sensitive to anti-HER2 agents. MBC patients with these tumors could be candidates for studies aimed at establishing chemotherapy-free regimens also in early metastatic disease.

Based on the evidence in the literature, there is definitely a subset of patients who may benefit from treatment with trastuzumab and lapatinib, particularly when given in combination. This research aims to evaluate prospectively the efficacy of trastuzumab or lapatinib as a single agent in a population of patients carefully selected for the presence of HER2 amplification in the tumor.

It is clear that an adeguate selection of patients for treatment with trastuzumab and either lapatinib could allow an optimization of available resources to ensure optimal patient treatment.

Nevertheless, a deeper understanding of the mechanisms of action of trastuzumab, especially action tied to antibody-mediated cytotoxicity (ADCC), may allow a better selection of patients based on molecular genetic markers predictive of response. In order to define activity and to identify biomolecular factors predictive of resistence to double HER2 blocked, a phase II study for untreated HER2 positive MBC patients with the use of trastuzumab plus lapatinib for 8 weeks continuing for further 8 weeks in case of response is ongoing [NCT00842998].

## Pan-HER tirosin kinase inhibitor: towards neratinib

In the recent years, a new group of compounds that bind irreversibly to the adenosine triphosphate binding pocket of HER receptors have been developed. One of these compounds, neratinib, has passed preclinical phases and is currently undergoing various clinical trials. Importantly, pan-HER TKI are very attractive strategies for this purpose. Among them, JNJ-28871063 has been shown preclinically to penetrate the blood barrier brain (BBB) more effectively than lapatinib, being also effective in improving survival of xenograft mouse models with HER2 positive intracranial metastases [50]. Neratinib, is another pan-HER TKI that is under clinical development fro HER2 positive BC which could prove highly effective also on central nervous system (CNS) metastases [51]. To date no date are evaluable about Neratinib activity in early recurrence brest disease. A trial in which the pan-HER inhibitor is compared with lapatinib plus capecitabine in HER2 positive patients previously treated with trastuzumab for MBC, is ongoing. Recently was presented preliminary results of randomized EXTENT study, in HER2 positive patients in which Neratinib was given after the completention of trastuzumab in adjuvant setting. The findings showed that Neratinib significally improved disease-free interval when compared with no further anti-HER2 adjuvant therapy [51]. After results of ongoing evidence of efficacy in second-third line therapy, probably neratinib could have an interesting position for first-line treatment in HER2 postive MBC patients.

## When the cancer expresses hormonal receptors

Hormonal therapy and trastuzumab represent one of the oldest and one of the newest treatment modalities for BC, respectively. Recent data have suggested that HER2 overexpression is associated with resistance to hormonal therapy and there is considerable preclinical evidence to support the existence of interaction or cross talk between HER2 and estrogen-receptor signalling pathways in BC. Preclinical data also demonstrate that adding trastuzumab to hormonal therapy results in greater antitumour activity than either agent alone. The existence of an inverse relationship between estrogen receptors ER expression and HER2 overexpression has also been well established clinically. Thus, a range of clinical trials are now ongoing to determine whether the addition of trastuzumab to hormonal therapy will provide BC patients with benefits in clinical practice. For patients with bone-only spreading disease and indolent disease progression, the combination of anti-HER2 and endocrine therapy as first-line treatment represents a valid therapeutic option. Three trials have examined the addition of HER2-targeted agents to aromatase inhibitors (AI) in postmenopausal women with a first recurrence (Table 1).

**Table 1:** Studies with anti-HER2 molcules plus hormonal therapy in the first-line metastaic breast cancer

Author	*N*. of patients	Treatment	PFS (months)	OS (months)	Response Rate (%)
Kaufman et al. 2009 (TANDEM study)	207	Anastrozole 1 mg po daily + T iv (4 mg/kg loading dose, then 2 mg/kg weekly)	4.8	28.5	21
		Anastrozole 1 mg po daily	2.4 (*p* = 0.002)	23.9 (*p* = 0.325)	7 (*p* = 0.018)
Huober et al. 2012 (ELECTRA)	92	Letrozole 2.5 mg po daily + T iv (4 mg/kg loading dose, then 2 mg/kg weekly)	14.1	NR	27
		Letrozole 2.5 mg po daily	3.3 (*p* = 0.23)	NR	13 (*p* = 0.002)
Johnston et al. 2009 (EGF30008)	263	Letrozole 2.5 mg po + Lapatinib 1500 mg po daily	8.2	33.3	28
		Letrozole 2.5 mg po + placebo po daily	3.0 (*p* = 0.019)	32.3 (*p* = 0.113)	15 (*p* = 0.021)

*PFS* progresson free survival, *OS* overall survival, *T* trastuzumab

In the TANDEM study, 207 patients were randomized to anastrozole (1 mg daily) plus trastuzumab (4 mg/kg loading dose, followed by 2 mg/kg weekly) or to anastrozole alone [52]. The combination arm was associated with an improvement in PFS (4.8 months *vs.* 2.4 months; HR 0.63) and an irrelevant improvement in OS (28.5 months *vs.* 24 months). Also the response rate was in favour of the combination therapy (20 *vs.* 7 %). The most common toxicities seen in the combination arm were fatigue (21 %), vomiting (21 %), and diarrhea (20 %); however, the vast majority of events were grades 1 and 2.

The combination of letrozole plus trastuzumab was compared with letrozole alone in the Electra study [53]; fifty-seven postmenopausal patients were randomized to receive letrozole (2.5 mg daily) with or without trastuzumab (4 mg/kg loading dose, followed by 2 mg/kg weekly). In addition, HER2-negative patients were enrolled as a third cohort and were treated with letrozole alone. The trial experienced slow accrual and closed early, before the planned 370 patients could be enrolled. Nevertheless, the addition of trastuzumab to letrozole was associated with a significant improvement in TTP (14 months *vs.* 3 months; HR: 0.67), the duration of which was similar to the duration achieved in the HER2-negative group (15 months). The rates of response and clinical benefit were 27 vs 13 % and 65 vs 39 %, respectively, in favour of both trastuzumab-containing arm.

A third study, EGF30008, compared the all-oral combination of letrozole (2.5 mg daily) and lapatinib (1500 mg daily) with letrozole alone [54]. Of the almost 1300 patients enrolled, about a quarter of them had hormonal receptors [HRs] and HER2 positivity. In that subgroup, the addition of lapatinib to letrozole was associated with a significant improvement in PFS (8 months vs 3 months: HR: 0.71) and response rate (28 vs 15 %). Overall survival was not significantly different (33 vs 32 months). As previously seen with lapatinib, diarrhea was significantly more common in the combination arm (grade 3 and 4 diarrhea: 10 vs 1 %).

On Table 2 are reported ongoing clinical trials evaluating new anti-HER2 molecules combined with hormone therapy in first-line setting. PERTAIN is a randomized phase II trial conducting since 2012, exploring the combination of an aromatase inhibitor with trastuzumab and pertuzumab vs an aromatase inhibitor with trastuzumab in first line treatment of HR positive/HER2 positive advanced disease in postmenopausal setting [NCT01491737].

**Table 2:** Ongoing studies of first-line treatments for HER2 positive metastatic breast cancer

Study	Arms of Treatment	Population	*N*. of patients	Obiectives of Study
Peruse (MO28047)	P + T + Tx	mBC HER 2+	1436	Multicenter clinical practice, single-arm phase IV study to evaluate the safety of the combination in real life world
Pertain (MO27775)	P + T + AI vs T + AI	mBC HER2+ HR+ Post menopausal status	250	Randomized multicenter open label phase II study designed to assess the efficacy and safety of P+ T + AI [anastrozole or letrozole) or T+ AI. Pts in either arm may also receive induction chemotherapy for up to 18 weeks at the investigator’s discretion.
Velvet (MO27782)	P + T + VNR	mBC HER2+	210	Randomized, multicenter open-label, single-arm with 2 cohorts of patients (cohort 1 = sequential infusion of P and T; cohort 2 concomitant P and T) designed to evaluate the efficacy and safety of P in combination with VNR
Metapher (BO29159)	P + T (s.c) + Tx	mBC HER2+	400	Multicenter single-arm phase IIIb study, designed to evaluate the safety and efficacy of the combination P + T subcutaneously + Tx

*P* Pertuzumab, *T* Trastuzumab, *Tx* Docetaxel or Paclitaxel or nabPaclitaxel, *VNR* Vinorelbine, *AI* aromatase inhibitor, *mBC* metastatic Breast Cancer, *s.c.* subcutaneous

The DETECT V/CHEVENDO trial is a randomized phase III study which aims to compare the combination of trastuzumab, pertuzumab and a chemotherapy drug (docetaxel, paclitaxel, capecitabine or vinorelbine) with the combination of trastuzumab, pertuzumab and hormonal therapy (tamoxifen, fulvestrant, letrozole or anastrozole). It is an ongoing trial currently recruiting participants [NCT02344472]. Finally, the phase II 1303GCC trial will compare trastuzumab in combination with pertuzumab alone vs trastuzumab, pertuzumab and eribuline vs trastuzumab, pertuzumab and hormonal therapy (anastrozole or fulvestrant) in locally advanced or metastatic BC affecting patients aged 60 or more [NCT02000596].

Although for patients with HRs/HER2 positive disease improvements in TTP or PFS were seen with the addition of anti-HER2 molecules to endocrine therapy, the gains are modest, and no study has demonstrated an improvement in OS. Hence, the use of such approaches has to be weighted against the significant benefit in PFS and OS seen when anti-HER2 molecules are combined with chemotherapy as outlined earlier.

## Is there a treatment of choice for early brain metastases from HER2 positive breast cancer?

CNS progression is a frequent phenomenon in trastuzumab-treated patients. Whether or not it reflects the lack of penetration of trastuzumab through the blood brain barrier or a higher propensity of HER-2 positive disease to spread into the CNS, it is reasonable to continue trastuzumab in the subgroup of patients who develop CNS metastases as the only site of disease progression, since increasing evidence suggests a benefit in terms of OS for patients continuing trastuzumab after receiving radiotherapy for intracranial disease [55, 56]. On the other hand, lapatinib has been shown to be active in CNS metastases of HER2 positive patients, although its role in this setting requires further testing in clinical trials [57].

An important challenge is the prevention of CNS metastases in HER2 positive metastatic disease For the first time, in the recent CEREBEL study [58] patients with HER2- positive MBC without brain metastases were randomized to compare trastuzumab-capecitabine versus lapatinib-capecitabine in order to evaluate the incidence of CNS metastases as first site of relapse.

Patients enrolled onto CEREBEL had similar population characteristics to those reported in other large prospective clinical trials of HER2-positive MBC that used a capecitabine combination [57, 59, 60] with the exception of the proportion of patients previously exposed to trastuzumab, with more patients who were trastuzumab naive in CEREBEL trial. The study was inconclusive for its primary end point and was unable to demonstrate the prevention of CNS metastases by lapatinib-capecitabine compared with trastuzumab-capecitabine. A better outcome of disease in the entire population was observed in the trastuzumab-capecitabine arm [58]. However, lapatinib-capecitabine efficacy may have been affected by previous exposure to a trastuzumab regimen and/or when treatment was given as first- or second-line therapy in the metastatic setting.

Recent findings, revealed intersting activity of T-DM1 in HER2 postive patients with brain metastasis, also in that case without any loco-regional treatment for CNS recurrences. Probably T-DM1 therapy might be able to prevent out-growth to macro-metastases. In addition, T-DM1 could be useful for patients with established brain metastases that qualify for primary systemic therapy, although WBRT is likely to become necessary eventually [61, 62].

Still, these results suggest that ongoing investigation of T-DM1 in brain metastases is warranted.

Recently an interesting finding has been highlighted with the use of neratinib. The phase 2 study NeferTT compared paclitaxel associated with trastuzumab or neratinib in a cohort of 479 HER2 positive patients. Despite the result in terms of PFS was not in favour of either treatment arms, in the group treated with Neratinib the incidence of symptomatic brain metastases was significantly lower (8 vs 17 %, RR0.48, *p* = 0.002), as well as a follow up of two years relapse of disease at CNS was much lower (16 vs 31 %, *p* = 0.0036) [63]. Neratinib could have a potential role in patients at risk for CNS metastatic events. To investigate further the efficacy of neratinib in metastatic ERBB2-positive breast cancer, the National Surgical Adjuvant Breast and Bowel Project (NSABP) Foundation has recently initiated a phase 1b/2 study of neratinib in combination with ado-trastuzumab emtansine as second-line therapy (NCT02236000).

Limitations of the research include the lack of specific data on patients with HER2-positive brain disease, how to measure efficacy of various chemotherapy agents and of anti-HER2 molecules. When there is a lack of multiple robust comparative studies, this precludes recommendations on the basis of high-quality evidence [64].

## The future landscape in first-line treatment for HER2 positive disease

In Table 3 are showed the ongoing randomized studies in first-line advanced disease.

**Table 3:** Ongoing clinical trials evaluating new anti-HER2 molecules combined with hormone therapy in first-line setting

Trial	Phase	Treatment	Primary Objective
PERTAIN	II	Trastuzumab + Pertuzumab + AI vs Trastuzumab + AI	PFS
DETECT V/CHEVENDO	III	Trastuzumab + Pertuzumab + CT vs Trastuzumab + Pertuzumab + HT^a^	Safety
1303GCC	II	Trastuzumab + Pertuzumab vs Trastuzumab + Pertuzumab + Eribulin vs Trastuzumab + Pertuzumab + HT ^b^	ORr

*AI* aromatase hinibitor, *CT* chemotherapy (docetaxel, paclitaxel, capecitabine, vinorelbine), ^a^HT: Hormonal Therapy (tamoxifen, fulvestrant, letrozole, exemestane or anastrozole); ^b^HT: Hormonal Therapy (anastrozolo or fulvestrant); PFS: Progression Free Survival; ORr: Overall Response rate

Recently, preliminary results on safety of IIIb PERUSE trial (NCT01572038) [65] were showed; the study is assessing the safety of first-line pertuzumab plus trasuzumab associated to investigator’s chosen taxane [docetaxel (45 %), paclitaxel (47 %), or nab-paclitaxel (6 %)] in routine clinical practice. To the first analysis, therapy was discontinued most often for progression disease (pertuzumab 7 %, trastuzumab 7 %, taxane 4 %) and for AEs in 4 % (each agent). The safety profile of the study is consistent with previous clinical experience of Cleopatra trial, and no unexpected safety signals were seen [65]. It is in course of enrollment a multicenter international single-arm, clinical practice study, commissioned by EMA, to evaluate the safety of the combination in the real life world.

PERTAIN trial (NCT01491737) is looking at combining pertuzumab with trastuzumab and an aromatase inhibitor to treat HER2/HR positive advanced BC; The trial want to find out how safe the combination of pertuzumab, trastuzumab and an aromatase inhibitor is and the activity of the double anti HER2 block with endocrine therapy in postmenopausal patients.

In order to test the introduction of different chemotherapic agents from taxane therapy in association with pertuzumab and trastuzumab, phase II VELVET study (NCT01565083) is enrolling patients receiving or the two anti-HER2 molecule as separate infusion or receiving pertzumab and trastuzumab in single saline infusion bag after the first cycle.

To date is not investigated the subcutaneous trastuzumab administretion in association with pertuzumab. METAPHER trial (NCT02019277) is a multicenter, international, single-arm post licence investigation that was designed in order to evaluate the safety and efficacy of the combination with pertuzumab plus trastuzumab subcutaneously associated to taxane.

## Conclusions

In HER2 positive disease the use of trastuzumab is well established. The strong recent data clearly recommend the early use of pertuzumab in combination with trastuzumab and paclitaxel or docetaxel in MBC and ongoing trial will define the integration in the adjuvant setting of these double HER2 inhibition. The mandatory use of this combination in first-line metastatic setting is regardless of the site of the disease concerned. Although we do not have today any results regarding the activity of pertuzumab in population that develops early brain metastases, there is prelimnary evidence of a delayed onset of the CNS spreading with the inclusion of pertuzumab treatment to the standard HER2 double biological therapy. An attrattive field of research would be the response of double block on CNS metastases at the first recurrence of disease.

Positive data support the combination of trastuzumab and endocrine agents in that case in which is not well indicated chemotherapy incuding agents, such as elderly patients, indolent progressive disease, absence of visceral crisis. Actually, we have no data to support the use of HER2 double block plus hormonal therapy in selective cases with first recurrence of disease.

Unfortunately, all the HER-2 positive MBC patients will eventually develop resistance to pertuzumab plus trastuzumab and taxanes, or to trastuzumab combined with endocrine therapy, even those who initially benefit from the treatment.

No other anti HER2 biological agents showed better efficacy than pertuzumab and trastuzumab in first-line therapy. T-DM1 seems to offer improved safety and efficacy over two widely used treatments for HER2-positive MBC. T-DM1-associated adverse reactions were generally manageable with appropriate dose modification and supportive care. The safety profile in first-line MBC or early BC is expected to be similar, and clinical trials are under way to evaluate T-DM1 in these settings.

There is an urgent need for prospective biomarker-driven trials to identify patients for whom dual HER2 targeting is cost-effective [66–68].

In conclusion, pertuzumab associated to trastuzumab represents now the milestone of first therapeutic approach in HER2 postive subtypes tumor, and the benefit in terms of outcome disease achieved with “Cleopatra treatment” is one of the more deep enthusiastic result obtained in the story of MBC cure.

Finally, the research has focused on molecular mechanisms underlying resistance to trastuzumab and pertuzumab. However, there is still much that we need to lern, in particular to better define activity correlated with biomarkers that could be define resistence or sensitivity to the specific target molecule.

The future is one in which it will be possible to use an ample armamentarium of combination therapies directed against HER2 and key signaling components integrated in the HER network. This therapeutic approach will allow clinicians to tailor the clinical management of the individual patient on the basis of tumor-specific biomarker profiles, as captured at the time of diagnosis and in the course of treatment.

## Abbreviation

BBB, blood barrier brain; BC, breast cancer; CNS, central nervous system; EMA, European Medicines Agency; FDA, Food and Drug Administration; HER2, human epidermal growth factor receptor 2; MBC, metastatic breast cancer; ORr, overall response rate; OS, overall survival; PFS, progression free survival; TK, tyrosine kinase; TTP, time to progression
